# The role of Medieval road operation on cultural landscape transformation

**DOI:** 10.1038/s41598-021-00090-3

**Published:** 2021-10-22

**Authors:** Michał Słowiński, Achim Brauer, Piotr Guzowski, Tomasz Związek, Milena Obremska, Martin Theuerkauf, Elizabeth Dietze, Markus Schwab, Rik Tjallingii, Roman Czaja, Florian Ott, Mirosław Błaszkiewicz

**Affiliations:** 1 Past Landscape Dynamics Laboratory, Institute of Geography and Spatial Organization, Polish Academy of Sciences, Twarda 51/55, 00818 Warsaw, Poland; 2 Present Address: GFZ -German Research Centre for Geosciences, Section Climate Dynamics and Landscape Evolution, 14473 Telegrafenberg, Potsdam, Germany; 3 Institute of Geosciences, University of Potsdam, 14476 Potsdam, Germany; 4 Institute of History and Political Sciences, University of Bialystok, 15-420 Białystok, Poland; 5 Department of Geoecology and Climatology, Institute of Geography and Spatial Organization, Polish Academy of Sciences, Twarda 51/55, 00818 Warsaw, Poland; 6 Institute of Geological Sciences, Polish Academy of Sciences, Twarda 51/55, 00-818, Warsaw, Poland; 7 Institute of Botany and Landscape Ecology, University of Greifswald, Soldmannstraße 15, 17489 Greifswald, Germany; 8 Alfred-Wegener-Institute Helmholtz Center for Polar and Marine Research, Research Unit Potsdam, Polar Terrestrial Environmental Systems, Potsdam, Germany; 9 Faculty of History, Nicolaus Copernicus University, W. Bojarskiego 1, 87–100 Toruń, Poland; 10 Department of Environmental Resources and Geohazards, Institute of Geography and Spatial Organisation, Polish Academy of Sciences, Twarda 51/55, 00-818, Warsaw, Poland

**Keywords:** Environmental economics, Environmental impact, Palaeoecology, Climate-change ecology

## Abstract

Connecting pathways are essential for cultural and economic exchange. Commonly, historians investigate the role of routes for cultural development, whereas the environmental impacts of historical routes attract less attention. Here, we present a high-resolution reconstruction of the impact of the major trade route via* Marchionis* in the southern Baltic lowlands on landscape evolution since more than 800 years. We combine precisely dated annually laminated sediments from Lake Czechowskie alongside via* Marchionis* and pollen data at 5-year resolution together with historical data. The transformation from a quasi-natural to a cultural landscape occurred in three phases (1) an early phase until the mid-fourteenth century with slowly increasing human impact. (2) an intensification of environmental disturbance until (3) the mid-nineteenth century when via* Marchionis* became a modern traffic route with strong environmental impacts. Superimposed on the long-term development were repeated interruptions by short-term downturns related to societal crisis and political decisions.

## Introduction

**Figure 1:**
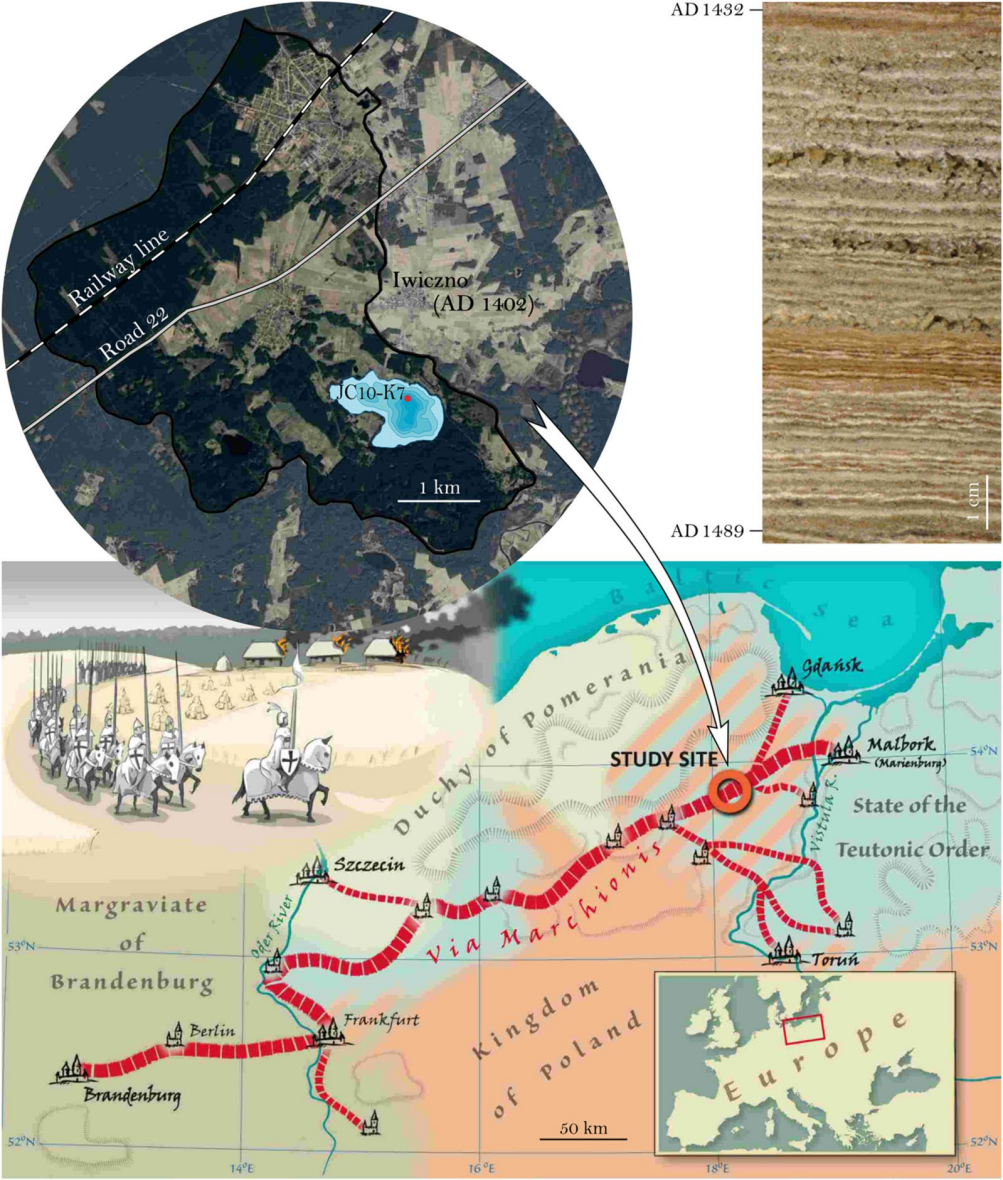
Present day location of Lake Czechowskie with coring site and catchment and modern road and railroad locations (upper left, image source: air image provided by Head Office of Geodesy and Cartography, Warsaw, Poland). Upper right: core photograph showing the varved sediment interval from AD 1489 – 1432. Bottom: Course of the via* Marchionis* and location of the lake (red circle) on a map which provide special to this manuscript by Piotr Kann.

Anthropogenic pressure on landscapes related to the growth and development of human population is reflected in long-term land use changes ^1^ with consequences for hydrological and erosional cycles at the local^2,3^, regional^4,5^ and global scale^6–8^. Land routes have significantly contributed to transform landscapes from natural to cultural and they were an important factor in the development of civilizations. Famous examples are the Roman roads, Silk Roads^9^ from China or the Amber Road in Central and Eastern Europe^10–12^. In history, we discern a multilayered and mutual relationship associated with the development of settlements and land routes^13^. However, we recognize also the negative impacts of mobility like the spread of plagues, for example the Black Death in mid-fourteenth century and later^14^. Investigating the impact of road construction and settlement network on local environments^15^ in great detail will contribute to a better understanding of the transformation of natural into cultural landscapes. One rarely applied approach to obtain such information is to investigate high-resolution lake sediment records located in the vicinity of a major route since lake sediments have been demonstrated to reliably record various aspects of past human impact^16,17^. The ultimate prerequisite for investigating lake sediments to trace environmental change on human or historical time scales is a precise sediment chronology and sufficient proxy data resolution^18,19^. We mainly utilize pollen and charcoal data at 5-year resolution as proxies for landscape openness and agriculture and µXRF element scanner data at sub-annual resolution as erosion proxy. The comparison of sediment proxy changes with historical events at great detail enables to investigate environmental impacts of historical events^18^. Ideal recorders of environmental change in the human habitat are annually laminated (varved) sediment records^19^ like Lake Czechowskie^20^ which we, therefore, have selected for our study. This lake is located in close vicinity to the via* Marchionis* (Fig. 1), which has been a main West–East connecting road in the southern Baltic lowlands since almost 800 years^21^.

The first existence of via* Marchionis* (VM) was confirmed in the thirteenth century (1286)^22^ as one of the branches of the route running from Frankfurt (Oder) to Pomerelia (Eastern Pomerania) (city of Gdańsk and the castle of the Teutonic Order in Malbork/Marienburg) which split into several smaller parts in western Poland^21,23–25^. For several centuries this route became a main pathway of migration (Fig. 1) and its particular importance is documented by its former terms – via* Regia Prussica* or via* Regia Nove Marchiae.* This implies that the rulers whose lands were crossed by the road were obliged to guarantee the safety of all travelers^26^. It was of particular importance during the development of the state of the Teutonic Order in the lands of Prussia, when troops regularly used this route to support the Teutonic Knights, first in the battles with the pagan Prussia “Holy War”, and in the following centuries in wars with Poland and Lithuania^27^. The importance of this road in the early modern period is evidenced by the fact that in 1524 the Polish king established custom points between its two branches to collect tolls from passing merchants^28^ and in 1549 the VM was described as “great and significant”^29^. Analysis of tax data revealed Skarszewy and Starogard economically thriving towns along via *Marchionis*, which would mean that in the sixteenth century this road still retained its importance and was an attractive trade route for many merchants of the time. In the early nineteenth century, the road became a major factor for the development of the regional economy. Therefore, it was modernized and adapted to modern traffic technologies in the years 1816–1823 through widening up to 5 m and rubble paving. Before, the road had an irregular course and was strongly dependent on weather conditions^30^. This route remained geopolitically important even in the late nineteenth century when the Prussian king Wilhelm IV built a railway line linking Berlin and Königsberg along the same track parallel to the road. Thereby, the VM eventually became a modern multiple transport route.

Lake Czechowskie (JC) is located in the Tuchola Pinewoods (TP), region one of the largest pine forest complexes in Northern Poland^31^. Pine forests grow on deposits of the glacial outwash plain formed during the last glacial period^32^. The lake is surrounded by forest, agricultural land and a recreational area. The regional climate is influenced by continental and oceanic air masses^33^. JC has been selected for this study because the historical route VM crosses through the catchments and because its sediments are annually laminated providing precise time control and contiguous sampling at 5-year intervals.

The transformation of landscapes to the present anthropogenic state was a complex process and influenced by several factors. One important but often underestimated factor is the establishment of routes due the transport of people, knowledge, technologies but also diseases. Since the role of routes is not yet understood in detail, the goal of this study is to investigate with an example the direct environmental impacts related to a major historical route. Therefore, we combine information from natural (lake sediments) and written sources including locations of the rural settlements, economic impacts or war occurrence and their consequences on the local communities. Recent models of the adaptation strategies show that the pre-industrial societies have coped with both climate changes and economic difficulties^34^. The southern Baltic lowlands is a crucial region to understand the development and expansion of a European identity^35^. In particular, the Medieval crusades played an important role for the development of societies and it is expected that local environments along the routes they used like the via* Marchionis* were especially impacted.

## Results and discussion

The proxy records from Czechowskie lake sediments demonstrate that the long-term landscape transformation from a quasi-natural to cultural state did not continuously occur but took place in three main phases, which were repeatedly interrupted by short-term setbacks, mostly due to wars.

### Low human impact phase (1000 AD until 1350 AD)

**Figure 2:**
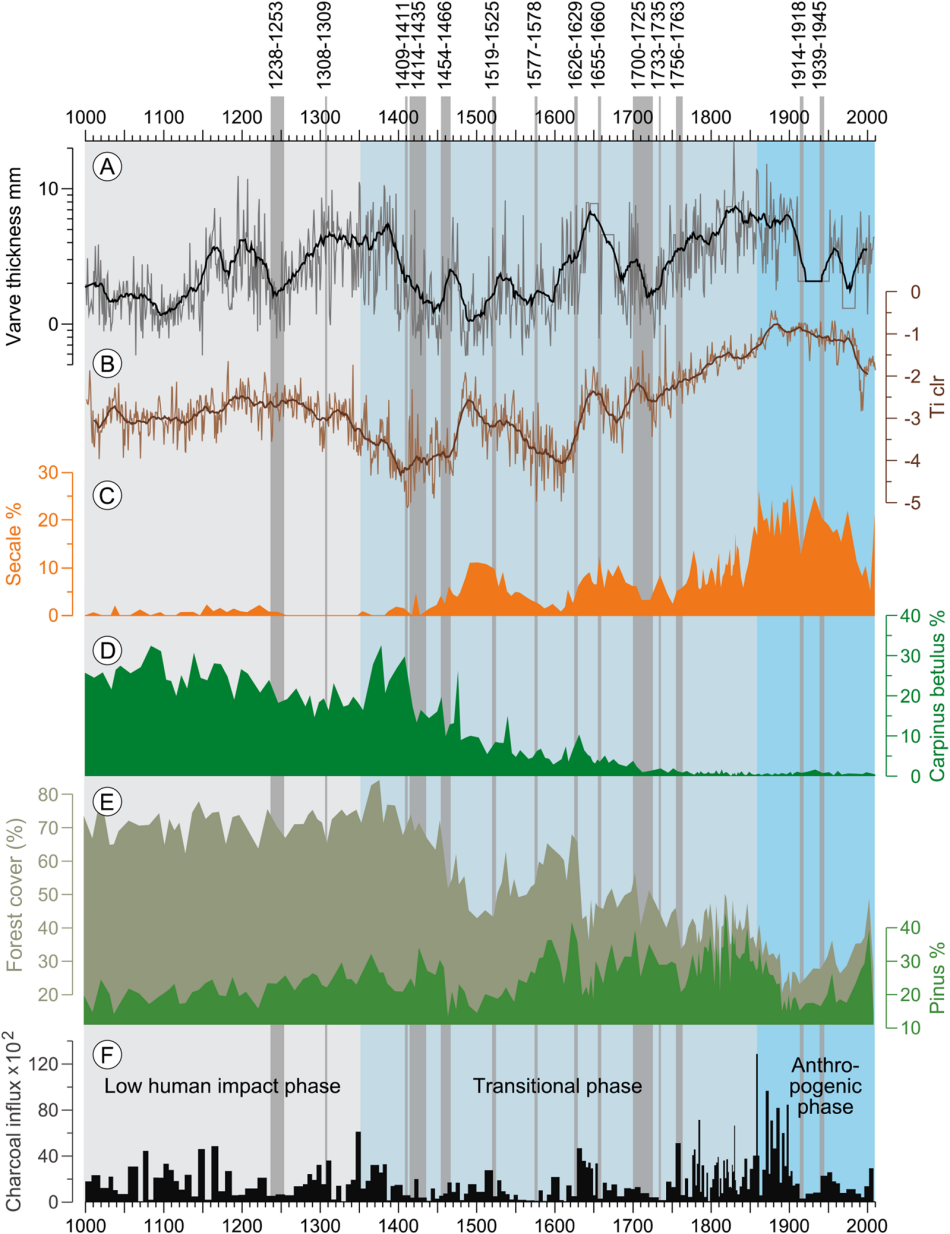
Sediment proxy data for the last 1000 years obtained from Lake Czechowskie cores. From top to bottom: (**A**) Varve thickness (21 running mean (bold line) and annual data; (**B**) Titanium (Ti) counts (clr) from XRF scanning at 50 mm resolution and 21 running mean (bold line) as proxy for detrital catchment material. (**C**) *Secale* % (indicator for agricultural land area), (**D**) *Carpinus* % (hardwood species used for buildings), (**E**) Forest cover % (proxy for land openness) and *Pinus* % (typical regional pioneer tree species) all pollen data are presented REVEALS transformation with the REVEALSinR function from the R package ‘disqover’ (Theuerkauf, et al.^57^ see methods). (**F**) Charcoal influx as proxy for fires. The grey bars indicate periods of wars in the region.

The environmental situation in the first phase was characterized by dominating mixed forest cover (65 up to 83% cover) with a large share of the *Carpinus betulus* (25 up to 32% cover) and *Pinus sylvestris* (up to 30% cover). Agricultural areas as indicated by *Secale* (rye) fields remained below 2% and, after a century with interruptions, became continuous only after ca 1150 AD (Fig. 2). This suggests low population density with only few and small settlements. During the wars 1238–1253 AD of the Teutonic Orders with Świętopełk II (Duke of Pomerelia) *Secale* farming abruptly vanished completely again. Most likely the troops of the Teutonic Knights devastated the area when they used the pathway along the lake. Although for this time there are no written documents proving the existence of the via* Marchionis* (VM)^36^, documents of the first towns from 1241 AD (Borzechowo) and 1305 AD (Zblewo) indicate that a connecting pathway already existed at that time. The disappearance of *Secale* fields is accompanied by an increase in pine and decreasing erosion rates which reflect immediate re-forestation in the vicinity of the lake lasting for about a century. Apparently, people did not settle again in this area for about an entire century after the wars. This is explained by the poor, mostly sandy soils around the lake, which were described as ’infertile’ (LMC 1565: 133–135) and unsuitable for intensive cultivation^37^ especially with the early medieval two-fields rotation system^38^. In morainic landscapes with more fertile soils in the political heartland of the Teutonic Order around Malbork (Marienburg) and Radzyń Chełmiński 100 km further north-east pollen data do not show such long-lasting absence of agricultural activity during the 14th and early 15th century^39,40^. The absence of agriculture around the lake might be further related to unfavourable climate in the first half of fourteenth century, which was characterized by precipitation extremes, floods and spring frosts^41,42^.

### Transitional phase (1350 AD until 1860 AD)

First evidence of re-appearing agriculture is seen around 1350 AD with a first peak in *Secale* and only two decades later continuous *Secale* cropping until today becomes evident (Fig. 2). The beginning of permanent agriculture marks the onset of the transitional phase of landscape transformation. The socio-economic preconditions for this development were already set nine years earlier with the implementation of the colonization under German law in Pomerelia from 1320 s-1340 s AD^43^. This reform of the German law was a significant turning point in the development of contemporary societies. The establishment of new legal order (the right of peasants to use the land and to pay tributes in the form of crops or rents) and the implementation of a completely new spatial organization of medieval villages (concentration of previously dispersed settlements) and occupying previously pristine areas enabled significant economic development at that time^44^. The institution of German law further favored technological changes including the open fields system that has contributed to accelerated deforestation, expansion of arable land and introduction of crop rotation^43,45–48^. Together, these developments fostered agriculture and forced major changes in the structure of the landscape (Fig. 2, S6). It should be noted that, on longer-term perspective, these developments^49^ might have contributed to later crisis in the economy of the late Middle Ages through the over-exploitation of arable land .

**Figure 3:**
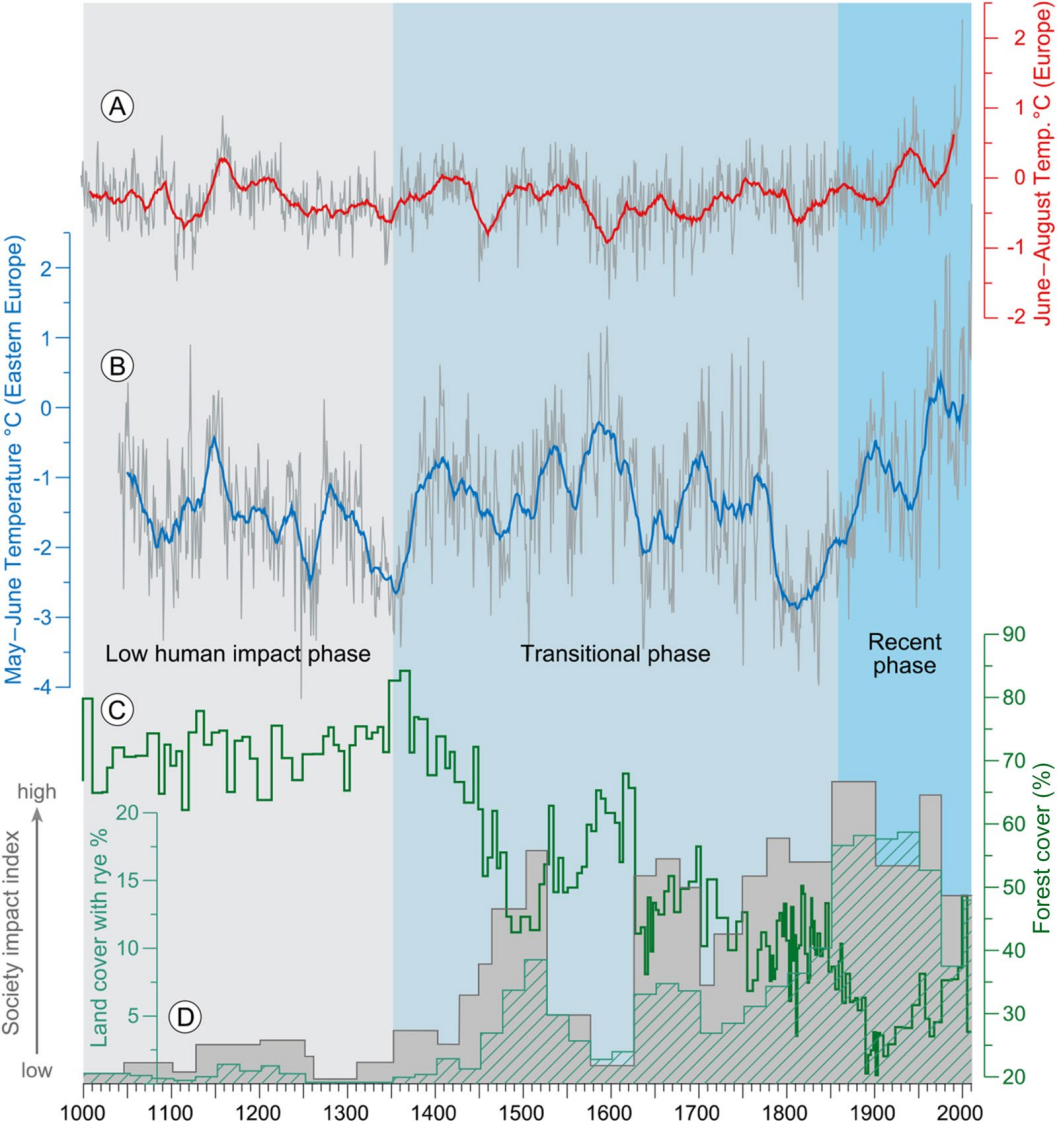
European summer June–August temperature reconstruction (**A**) anomalies with respect to reference period AD 1500 – AD 1850) from^42^; (**B**) Eastern European May–June temperature reconstruction from tree rings (deviation from mean of reference period 1961–1990)^41^; (**C**) Forest cover calculated from arboreal pollen (AP) with the REVEALSinR function from the R package ‘disqover’ Theuerkauf, et al.^57^; (**D**) Society Impact Index for 50-year means (grey bars) derived from % land cover with rye (green dashed bars) and historical information (see supplement).

The new legislation occurred at a long period without warfare in the region and favorable climatic conditions with increasing spring and summer temperatures (Fig. 3) in the second half of the fourteenth century^41,42^. Furthermore, a suitable infrastructure for mobility was provided by the existence of the VM. In combination, these favorable socio-economic and climatic conditions paved the way for an accelerated colonization involving the construction of administrative urban sites and military fortresses as well as the development of the rural background with the foundation of new villages like, for example, Iwiczno in 1402^50^, located only 500 m north of Lake Czechowskie. This rural development is well expressed in the re-appearance of *Secale* farming with a simultaneous *Carpinus betulus* and total arboreal pollen decline (Fig. 2).

The period of growth and economic development was abruptly interrupted by two wars between Poland and the Teutonic Order in 1409–1411 and 1414–1435 AD which left a clear imprint on the landscape as documented in vegetation setbacks and decreasing erosion (Fig. 2). The Pomeranian and Polish Crown armies recruited mercenaries *e.g.* from Czech territories or the western border of the margrave who used the VM and damaged villages along the route^30^. One typical example has been reported from 1433 AD when auxiliary troops of the Czech Hussites passed by the Neumark province on Pomeranian and burned and pillaged the settlements of the Teutonic Order lieges^30^. These events of destruction caused population declines and suppressed farming activities for about two decades before the fields recovered (Fig. 2), only briefly interrupted again by the Thirteen Year War (1454–1466 AD).

The peace treaty between the Teutonic Order and the Kingdom of Poland in 1466 AD heralded a period of peace for more than 50 years which favored rapid economic development and intensification of agriculture^36^. This is clearly expressed by the four-fold increase of *Secale* fields reaching a distinct maximum of > 11% cover in less than 40 years. This remained the highest share of *Secale* crops for 350 years in this region. The rapid expansion of agricultural land at the expense of forest areas is well-illustrated also by the decline in arboreal area from ca 70% to 50% cover. For the first time, we also observe consequences of increasing industrial activities. The rapid decline in the hardwood species *Carpinus betulus* was caused by exploitation for building material and especially for the production of charcoal and potash^30^. The resulting deforestation had a direct impact on catchment erosion leading to a higher detrital influx into the lake (Ti, Fig. 2). Again, the VM was an essential factor for the regional development because it was the main pathway crossing Pomerelia , leading to the two largest city ports of Gdańsk and Elbląg at the Southern Baltic cost during this time. The development of these towns of the Hanseatic League stimulated the development of the entire region including smaller villages along the VM near Lake Czechowskie like Zblewo and Bytonia^43^. After 5 decades of agricultural expansion and landscape opening the next war between the Teutonic Order and Poland from 1519 to 1525 AD again led to regression as evidenced by the major drop in *Secale* crops by 50% and reforestation as indicated by the rise of pioneer tree species *Pinus* and *Betula* from 45% up to 70% cover (Forest cover %, Fig. 2). Reforestation in turn caused an immediate decrease in catchment erosion (Fig. 2). This direct impact on the landscape is related to the destruction of villages along the VM by military troops of about 2,000 rides and 8,000 infantry, which set off from the Margraviate of Brandenburg to help the Teutonic Order^51^. The AD 1519–1525 war marks the onset of a longer decline with a 90% cover reduction of *Secale* lasting for about 80 years, probably enhanced by three waves of plague in 1538, 1549 and 1564 AD^52^. The devastation is expressed in the description of the villages Iwiczno and Piece located in the Czechowskie catchment as "Pustkowie" – a literally deserted area^30^.

Re-development of agriculture is expressed by a rapid increase in *Secale* and sharp decline in tree pollen that coincided with an increase in erosion at around 1610 AD. The agricultural development was only briefly interrupted by the wars with Sweden in 1626 – 1629 AD and 1655 – 1660 AD which apparently did not strongly affect the region around Lake Czechowskie. The next massive decline in agricultural activity is related to the Third Northern War from 1700 – 1725 AD that led to an abrupt fall in *Secale* fields of 50% cover from 6.2 to 3.2% cover within 5 years, resulting in a brief period of reforestation and decrease in catchment erosion (Fig. 2). The negative impact of this war most likely was further reinforced by unfavorable climate conditions related to the Late Maunder solar Minimum^34,41^. Very cold years that caused bad harvests and diseases like, for example, the rinderpest in 1709 and 1734^52,53^ exacerbated the situation for agriculture. This disturbance of landscape evolution lasted for a period of six decades that suffered from three consecutive wars. Immediately after the last of these, the ‘Seven Years War’ (1756–1763), crop area rapidly increased to 14% cover again. A re-intensification not only of agricultural and livestock farming activities but also of protoindustrial activities like smelters is documented by the Royal Lands Review from 1765^54^. The first partition of Poland in 1772, when Pomeralia was annexed to Prussia led to changes in land management, in particular, the introduction of pine monocultures in silviculture and the drainage of wetlands^31^. Especially the planting of pine monocultures caused higher frequency of forest fires as evidenced in an increasing amount of charcoal in the sediments (Fig. 2)^31,55^.

### Anthropogenic-dominated phase (1860 AD until today)

The onset of the final, anthropogenic-dominated phase of landscape transformation is difficult to exactly define. An obvious option is around 1860 when we observe the strongest increase in *Secale* up to almost 30% cover (Fig. 2). This was related to industrial revolution in the larger region and especially the growth of port cities in the vicinity of the Gdańsk Bay (~ 50 km) with growing demands for agricultural products. The industrial revolution began in the 1860s and 1870s and resulted in a more perforated landscape with a denser settlement structure and increase of the agricultural areas documented in cartographic maps^30,31^. A major change point for landscape transformation was the extension of the road by the construction of a parallel railway in 1871 in order to link the Prussian capital Berlin with the eastern province (city of Königsberg). This new transportation system provided new possibilities for developing the region and facilitated local enterprises such as sawmills^16^. The construction of the railway further accelerated the export of wood from the Tuchola Forest^16^. This is well documented in the rapid decrease of pine forest from ca 30% to 15% cover in only 23 years (1873 – 1896). This decrease in forest cover caused a peak in catchment erosion (Fig. 2). Another consequence of the operation of the railway with steam engines was the drastic increase of forest fires in the region as reflected by a peak in the charcoal record (Fig. 2)^55^. Significant reforestation in the region mainly with pine (Fig. 2) occurred only after World War II leading to a decrease in catchment erosion^31,55,56^.

## Materials and methods

### Historical data

The basic settlement structure was developed on the basis of existing literature and available databases for the sixteenth century (Supplement part A: historical information). In economic analyses we used the king’s estates inventories from 16th to 18th  centuries^54,58,59^ that provide detailed information on the economic situation of rural settlements around Lake Czechowskie with the buffer of 20 km over a period of three centuries. Economic inventories gave further information about the use of natural resources in the region. We complemented this information source with other materials, including royal documents^60^. The available written materials focus on the period from 1600 to 1800 for which abundant historical data is available.

## Lake Czechowskie varved sediments

Lake Czechowskie (JC) is located about 1,5 km south of the VM in Northern Poland (53° 52.2’ N, 18° 14.1’ E, 108 m a.s.l.) and has a surface area of 0.73 km^2^ and a maximum water depth of 32 m^61^. Today, the lake is surrounded by forest, agricultural land and a recreational area. The VM crosses the lake catchment (19.7 km^2^) in its northern part. The sediments of the lake are seasonally laminated and proven as calcite varves by sediment trap studies^62^ which predominantly consist of autochthonous material with only minor contributions of detrital catchment material^20^. Varve boundaries have been microscopically defined for each varve. Microscopic analyses revealed that the varves consist of a seasonal succession of three sublayers commencing in spring with (1) diatom blooms followed by (2) endogenic calcite formation and a final re-suspension layer in autumn that in some years is accompanied by a second diatom bloom (Supplement part B: sediment analyses). A robust chronology has been established based on microscopic varve counting, identification of the Askja AD1875 volcanic ash layer, AMS 14C dating of terrestrial plant remains and 137Cs activity concentration measurements^20,63^ (Supplement part B: sediment analyses). The excellent varve preservation and mean varve thickness 0.6 -22.4 mm/yr enabled precise sampling in 5-year (varve) intervals for high-resolution pollen analyses.

## Proxy interpretation

In order to obtain quantitative vegetation reconstruction we applied the REVEALS model^64^ with the REVEALSinR function^57^ (Supplement part B: sediment analyses). A key indicator taxon for agriculture practice is *Secale* pollen (rye) (Fig. 2), while the sum of non-arboreal pollen from terrestrial plants reflects deforestation (Fig. 2. and Fig. S6.). In addition, individual tree taxa allow tracing economic development and forest management practices. For example, Hornbeam (*Carpinus*) was a major hardwood species for economic development during medieval times and mirrors cultural and economic growth in the region. Pine reflects developments in forest management especially since the late eighteenth century^65^. Variations in titanium, measured at sub-annual resolution are a proxy for detrital sediment transport from the catchment into the lake which is interpreted as a measure for erosion processes triggered by anthropogenic changes in vegetation cover.

### Societal impact index (SII)

To provide an improved measure for anthropogenic pressure on the landscape, we developed a Societal Impact Index (SII, Fig. 3) through combining quantitative and continuous paleoecological data (% land cover with rye calculated with the REVEALS model) with qualitative and non-continuous historical data (e.g. reports about foundation of towns, population growth or decline, infrastructure, wars, plagues, etc.). The land cover data with rye have been averaged to 50-year mean values (supplementary information) and form the basic part of the index. These values then were increased/decreased based on subjective evaluation of historical documents from the corresponding time intervals. Despite the subjective component, we consider this approach more suitable to depict anthropogenic pressure on the landscape than paleoecological data or historical information alone.

### Data availability

All data needed to evaluate the conclusions in the paper are present in the paper and/or the Supplementary Materials. Additional data related to this paper may be requested from the authors.

## Conclusions

We demonstrate that high-resolution pollen and sediment proxies from varved lake sediments evaluated together with written records allow to trace landscape transformation during the last 1000 years from a quasi-natural to the modern anthropogenic state at great detail. This transformation was not a steady process but characterized by several setbacks and periods of acceleration caused by complex interactions of socio-economic and climatic factors. The via* Marchionis* played a large but also diverse role in the process of landscape transformation during the last 800 years. On one hand, the route favored the spread of new ideas and technical developments and enabled people to move into the area and, thereby, favored the growth of regional economy. On the other hand, this route was repeatedly used for troop movements during wars leading to local destruction, population decreases and setbacks in agricultural use of the landscape. Our data also shows that the effects of major political decisions like, for example, the agrarian reform in AD 1343 can have a clear impact on the environment even if that may be delayed by several years.

## Supplementary Information

Supplementary Information.
