# Supplementary material for: The role of Medieval road operation on cultural landscape transformation
Source: Sci Rep. 2021 Oct 22;11:20876. doi: 10.1038/s41598-021-00090-3 (PMC8536699; doi:10.1038/s41598-021-00090-3)
Supplement: Supplementary file 1 — Supplementary Information. [file 41598_2021_90_MOESM1_ESM.docx]

Supplementary Information

**The role of Medieval road operation on cultural landscape transformation**

Słowiński et al.

**Supplement part A: Historical information**

### Development of the settlement structure in Middle Ages and Early Modern period

The 16^th^ century is the first moment in the medieval and modern Polish history for which it is possible to reconstruct an almost complete settlement network in territories belonging to the Kingdom of Poland ^1^. What is important here is the source base enabling historians to reconstruct the location, size and demographic and economic potential of individual settlements. The sources include tax registers and reviews of royal, ecclesiastical and municipal lands. There are two reasons for basing our economic and environmental analyses mostly on data from the reviews of royal lands from different periods. First, they are fully comparable due to uniform (or nearly identical) land description forms. Second, in comparison with other sources, reviews of royal lands are relatively well preserved. Almost complete records have survived for the years 1565, 1624, 1660 and 1765 ^2, 3^. Therefore, our detailed analyses focus on the period between Middle Ages and the end of the 18^th^ century. We have limited the study area to 20 kilometers around the center of Borzechów royal estates located by Lake Czechowskie ^4, 5^ (Fig. S1). Written sources, such as Ibrâhîm ibn Ya`qûb’s description of his journey across Europe or Life of St Adalbert, show that before 1000 AD there were only a few small self-sufficient communities in Pomerania, which did not form any kind of wider political union. Around the turn of the 10^th^ century, the whole region was united by the Piast dynasty and Christianity was introduced ^6^. During the period of feudal fragmentation in Poland, the Pomeranian dynasty of the Samborides grew in power and contributed to the development of the area under study in early 13^th^ century. The second half of the 13^th^ century was the time of domestic unrest in Eastern Pomerania, political and territorial conflicts with the Teutonic Order, and Polish raids led by dukes from the Piast dynasty. One of the parties in the struggle for political control over Eastern Pomerania was Brandenburg, whose forces took part in the siege of Gdansk in 1271. They must have used via Marchionis in order to reach Pomerania. Military operations led to the area’s destruction and it was not until its eventual conquest by the Teutonic Order in the 14^th^ century that it started developing again.

The development of more advanced settlement structures occurred later in the second half of the 14^th^ century. We associate this change with a wide-ranging settlement programme based on the German law as well as with the development of road infrustructure, e.g. via Marchionis, and, indirectly, also with the increasing economic position of Pomeranian towns. This development was connected with intensive colonization involving the construction of a network of strong administrative and military points, e.g. castles, and the development of their rural supply base ^7^. Colonization of this territory was accompanied by the introduction of new village organization and new farming methods. The peasants were apportioned large farms (16.8 ha and larger) which, thanks to the use of modern tools (heavy plough, iron harrow) and the introduction of a three-field cultivation system (open fields system), satisfied not only the needs of the peasant family, but also produced for sale. In each village there were also large, market-oriented farms of village leaders (45 ha or more).

On the basis of existing historiography of human settlement in the area under study ^4^ the number of settlement units around the year 1570 was established to be 83, including one castle in Kiszew, one town in Starogard Gdański, one inn in Złe Mięso, one deserted settlement in Iwiczno, and seven manorial estates (The map of Polish lands of the Crown in the 16th century; on-line: http://atlasfontium.pl/index.php?article=korona) ^4^. All other settlements were villages. In the following centuries, the number of rural settlements in this area did not change significantly, and the existing ones were making use of local natural resources ^5^.

### Roads - via Marchionis

The north-western part of the area under study was crossed by via Marchionis, whose existence was confirmed in the 13^th^ century ^8^. It was one of the branches of the route running from Frankfurt (upon Oder) to Pomerania, which in turn split into several smaller parts in western Poland ^9^. According to the system of classifying communication routes at that time, this road was considered a public road (via Regia Prussica, via Regia Nove Marchie). This would imply that the rulers whose lands were crossed by the road were obliged to guarantee the safety of all its users ^10^. The importance of this road in the Early Modern period is evidenced by the fact that in 1524 the Polish king established customs points between its two branches, which collected tolls from passing merchants ^11^. In 1549 via Marchionis was described as “great and significant” ^12^. Thus, we treat it as a significant trade route which also played an important role in local communication ^13^. A road of this importance should therefore be treated first and foremost as an entity embedded in a wider network of relationships, consisting of a mass of local connections. It is possible to look at the potential of urban centres in Royal Prussia at the end of the 16^th^ century through data concerning the amount of tax paid on beer production in the towns of the region ^14^ as well as data concerning population. About 37% of population of Pomorskie voievodship lived in Gdańsk and 15 small towns. Population density was 11.4 people per 1 km^2^ ^15^. The area we studied was located on a line connecting German lands (Brandenburg), Central Poland (Greater Poland, Kuiavia) and one of the most important Baltic ports at that time - Gdańsk. The analysis of tax data revealed the existence of two economically thriving towns along via Marchionis - Skarszewy and Starogard (Fig. S2.). This would mean that in the 16^th^ century the road still maintained its importance and was an attractive trade route for many merchants at that time.

**Forests**

As regards the forests, surviving documents make it possible to reconstruct their types in some points of the area under study at the turn of the 16^th^ and 17^th^ centuries ^16^. They were predominantly mixed forests (dominated by pine trees), growing on very poor, sandy soils. The review documents reveal that the area immediately by the lakes near Borzechów was overgrown with dense thickets (LMC 1565: 137). In the early 17^th^ century, the policy of protecting the royal forests in the Tuchola Forest as well as in other royal estates in Pomerania was introduced. In 1614 queen Constance issued a document in which she prohibited deforestation of areas belonging to the royal domain. One of the entries in the 1624 review of royal lands contains a reference to the implications of this order. The reviewing officials noted that 70 years earlier tar making had been an important source of income for the county, but at the beginning of the 17^th^ century it was abandoned due to restrictions on tree felling (LWPK 1624: 231)^17^. Additional information is provided by onomastic material. Leopold von Schrötter's (1802–1812) map of Prussia shows settlements whose names were probably derived from the names of trees (beech, lime, oak) in deciduous forests nearby.

### Arable land and agricultural production

The soils around Lake Czechowskie were described in 16^th^-century reviews of crown lands as "poor", "infertile" and "sandy" (LMC 1565: 133-135) ^18^. The production structure of large farms and manor farms existing in this area indicates that rye was the dominant crop, accounting for 40–50% of the harvest, while oats were the most important of the remaining cereals. Other cereals - especially those requiring both better soils and intensive fertilization (barley, wheat) - were grown only occasionally (LWP 1565: 141) ^18^.

Among the settlements existing in the analyzed area, we examined 23 royal villages, for which historical sources provide basic economic data. During the colonization of the area in the later Middle Ages (from the middle of the 14^th^ century to the end of the 15^th^ century), a limit on the amount of land to be turned into peasant farms was introduced (Fig. S3). It was 1160 włóka chełmińska (Lat. mansi Culmenses), with one włóka being the equivalent of about 16.8 hectares. In the following centuries, the total amount of land in peasants’ hands in the 23 villages under study tended to diminish (Fig. S2). Due to the rise in the number of deserted farms and peasant fields and the simultaneous increase in the importance of grain exports, manor farms - i.e. large, market-oriented farms managed by royal officials - were established. In 1565 there were six of them in the territory under study and it can be assumed that their size was growing at the cost of peasant farms until the middle of the 17^th^ century. In the second half of the 17^th^ century a several-fold decrease in the area of land cultivated by peasants is documented. The grain production of existing and newly established manor farms was at a minimum level, which would only allow to produce enough food for the owner and hired laborers. However, the royal lands review of 1765 contains information confirming the revival of both peasant and manorial economy. It states that peasants in 23 villages cultivated 20% less land than in about 1500, but it was still four times more than after the Deluge. At the same time the number of manor farms increased to fifteen.

Reviews of crown lands reveal that in the vicinity of the village of Osieczna, tar production developed in the first half of the 17^th^ century and continued until the turn of the 18^th^ and 19^th^ centuries. The presence of glassworks in the area may also indicate that surrounding forests were intensively used as a source of firewood (LPK 1765: 83-84).

**Societal Impact Index (SII)**

This index was developed to provide a semi-quantitative measure for human impact since the beginning of the Middle Ages in the region around Lake Czechowskie. The numerical basis of the index was the land cover of secale calculated with REVEALS based on Czechowskie pollen data for 50-year time intervals These values were modified on grounds of historical data for those intervals for which such information was available (indicated in Fig. 3). We grouped historical documents in (1) those who reflect an increase in human activity and (2) those who suggest a decrease in human activity. Typical examples for (1) are foundations of towns (Table S2), geopolitical changes favouring socioeconomic growth, reports on new infrastructure, rising population, And, for (2) damages by wars and troop movements, plagues, and associated population declines. The rating of these socio-economic information has been subjectively decided by an interdisciplinary team of historical and environmental scientists. According to the rating of these information the index derived by secale land cover was up- or downgraded. We are aware that this approach combining quantitative proxy data and qualitative historical information might be biased by subjective evaluation and the SII thus is a qualitative measure valid only for the study region. However, through the combination of data it provides a better estimate of anthropogenic pressure on the landscape than paleoproxy data or historical information alone.

**Supplement part B: Sediment analyses**

**Lake sediment coring**

Four parallel and overlapping series of long cores were obtained using an UWITEC piston corer (90 mm diameter) in 2009 and 2012 from a floating platform from the deepest part (32 m water depth) of Lake Czechowskie. These cores are labelled as JC09-A, -B and JC12-C, -D with the longest core reaching 1346 cm. In order to obtain an undisturbed sediment-water interface several short surface cores were taken with an UWITEC corer from the deepest part of the basin. Each core was cut lengthwise in two halves, described, photographed and stored in core storage at 4°C. From surface and long sediment cores the continuous master composite profile JC-M2015 has been established by means of distinct macroscopic and microscopic marker layers. For this study, we investigate the uppermost 310 cm of the master composite that include cores JC10-K2 and JC09-A1, A2 and B1

**Varve analyses and counting**

Continuous varve and seasonal sublayer analyses has been carried out on a continuous series of 38 overlapping large-scale thin sections (10 cm long, 2 cm wide) using a transmitted-light petrographic microscope (Zeiss Axiophot). Thin sections have been prepared from fresh sediment after freeze-drying according to the method described by Brauer and Casanova ^19^. For qualitative analyses magnifications of up to 100x were used, while varve counting and sublayer thickness measurements were performed with 50x magnification.

The entire study interval is continuously finely laminated and interpreted as annual laminations (varves) consisting of three main sublayers. The seasonal succession commences with a diatom bloom in early spring after the ice break-up, followed by biogenic induced calcite precipitation of different grain sizes in late spring early summer. The third sublayer is formed by mixed sediments resuspended from shallower parts of the basin during wind and wave activity in fall. This seasonal succession observed in thin sections has been confirmed by sediment trap studies ^20^.

**Sediment chronology**

The chronology of the entire sediment record is constructed by a multiple dating approach based on varve counting supported by tephrochronology ^21^ and radiocarbon dating of terrestrial macro-remains. The chronology of the last 140 years has already been described in detail ^22^ including additional ^137^Cs activity concentration measurements which further confirmed the annual nature of the laminations. This study comprises the upper 310 cm of the composite record and the chronology is based on varve counting including the AD1875 Askja tephra originating from Iceland as anchor point ^21, 22^. Varve boundaries have been microscopically defined for each varve. For most of the study interval varve preservation was excellent except for some intervals with moderate to poor varve preservation from 151-131 cm, 84-79 cm, 26-18 cm and 11-9 cm. The uppermost two of these faintly varved intervals fall within the last 140 years and counting even of these intervals have been proven reliable by the Askja AD1875 tephra. The counting uncertainty has been determined by double counting of 7350 continuous varves from present day back in time. Double counts have been partly performed by the same investigator and partly by different investigators. The resulting difference between the two counts of 2% is in the typical range of varve chronologies obtained from comparable varved sediments ^23^. Therefore, we accept and apply an uncertainty of 2% also for the study interval as a conservative error estimate.

**Radiocarbon dating**

In total, 30 samples of terrestrial organic remains have been AMS 14C dated from the entire sediment record (unpublished) at the Poznań Radiocarbon Laboratory. Nine dates have been obtained from leaf and leaf fragments (8 samples) and *Carpinus* seeds (1 sample) in the study interval. Two of these dates have been omitted during the age modelling because they revealed too old ages (Table S4). Age modelling has been performed using the *P_Sequence* deposition model with a variable poison (k) parameter implemented in OxCal v.4.2 ^24, 25^.

**Major element scanning**

Split sediment cores have been scanned for their major element variations using the Itrax core scanner (COX Analytical Systems, Sweden) at GFZ Potsdam. Analytical resolution was 200 µm, i.e. at sub-annual scale, and an exposure time of 10 s was applied. In order to minimize the effect of varying sediment structures and organic contents the raw intensities were centre logratio (clr) transformed ^26, 27^. In this study, we only show variations of titanium as a proxy for detrital catchment material in the sediments.

**Sampling for pollen analyses at 5-varve resolution**

The macroscopically well visible varve structure allowed us to subsample the sediments in equal time interval of 5 varves/years in order to achieve sub-decadal resolution for pollen and charcoal data for precise linking with historical information. The sampling process was controlled by large-scale scans of the thin sections allowing to directly transfer varve counting results to each pollen sample.

**Vegetation reconstruction and sub-sample preparation**

Pollen samples (171 samples) were collected from the laminated sediments with 5 years steps resolution. Pollen samples were prepared using standard laboratory procedures ^28^ and using Lycopodium marker. Microscopic charcoal particles (size: > 10 μm) were counted from the same slides as pollen.

**REVEALS** was applied with the REVEALSinR function from the R package ‘disqover’ ^29^. Analysis includes the 21 most abundant upland tree and herb pollen types. Fall speed of pollen and pollen productivity estimates are taken from the PPE.MV2015 data set (Tab. S5)^30^. REVEALS was applied with the LSM dispersal model, adjusted for unstable atmospheric conditions. The 21 taxa are assumed to have covered 100% of the landscape.


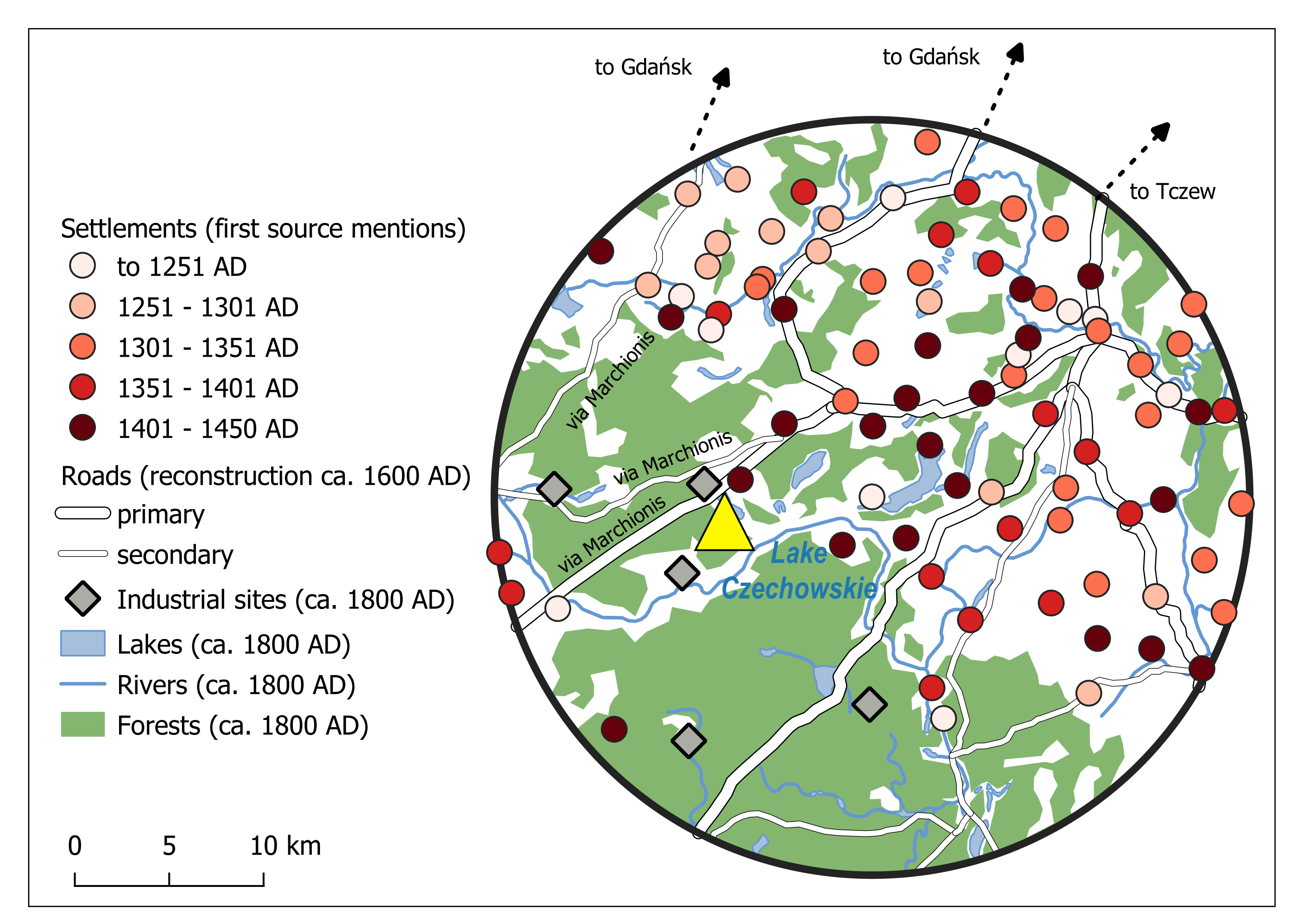


**Fig. S1. The historical case study buffer with 20 km distance radius.**


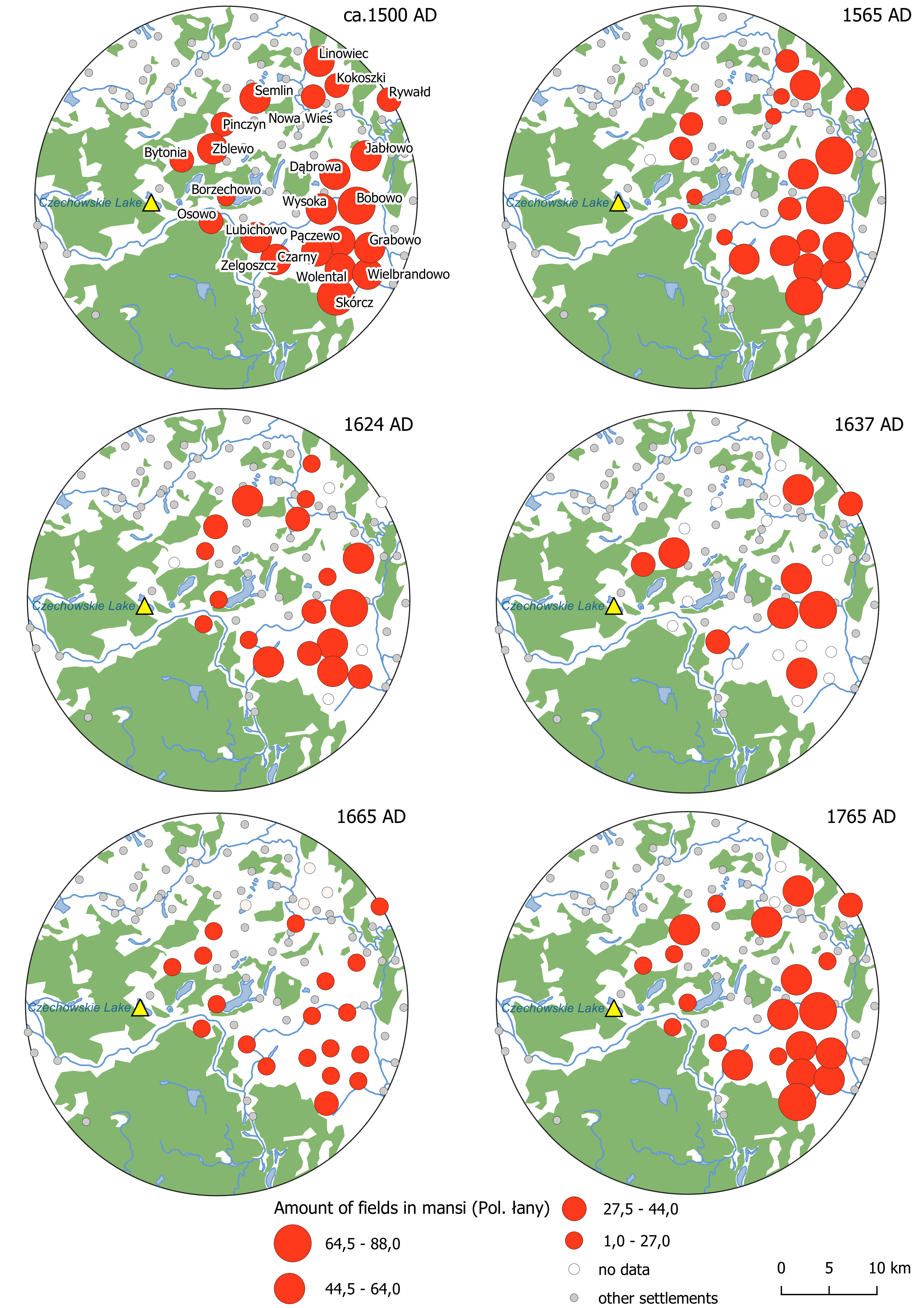


**Fig. S2. Changes in acreage of arable land in royal lands by Lake Czechowskie between ca. 1500 AD and 1765 AD.**


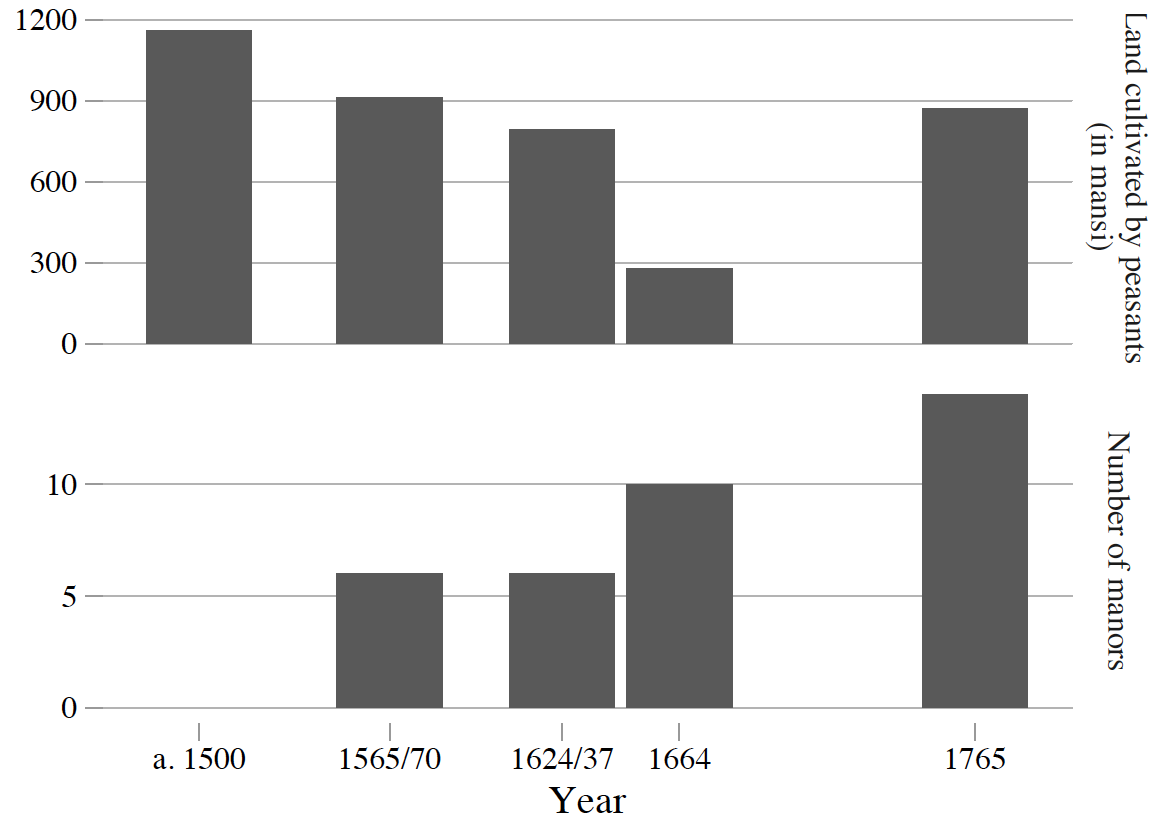


**Fig. S3. The correlation between amount of cultivated land and the development of the manors in the case study area.**


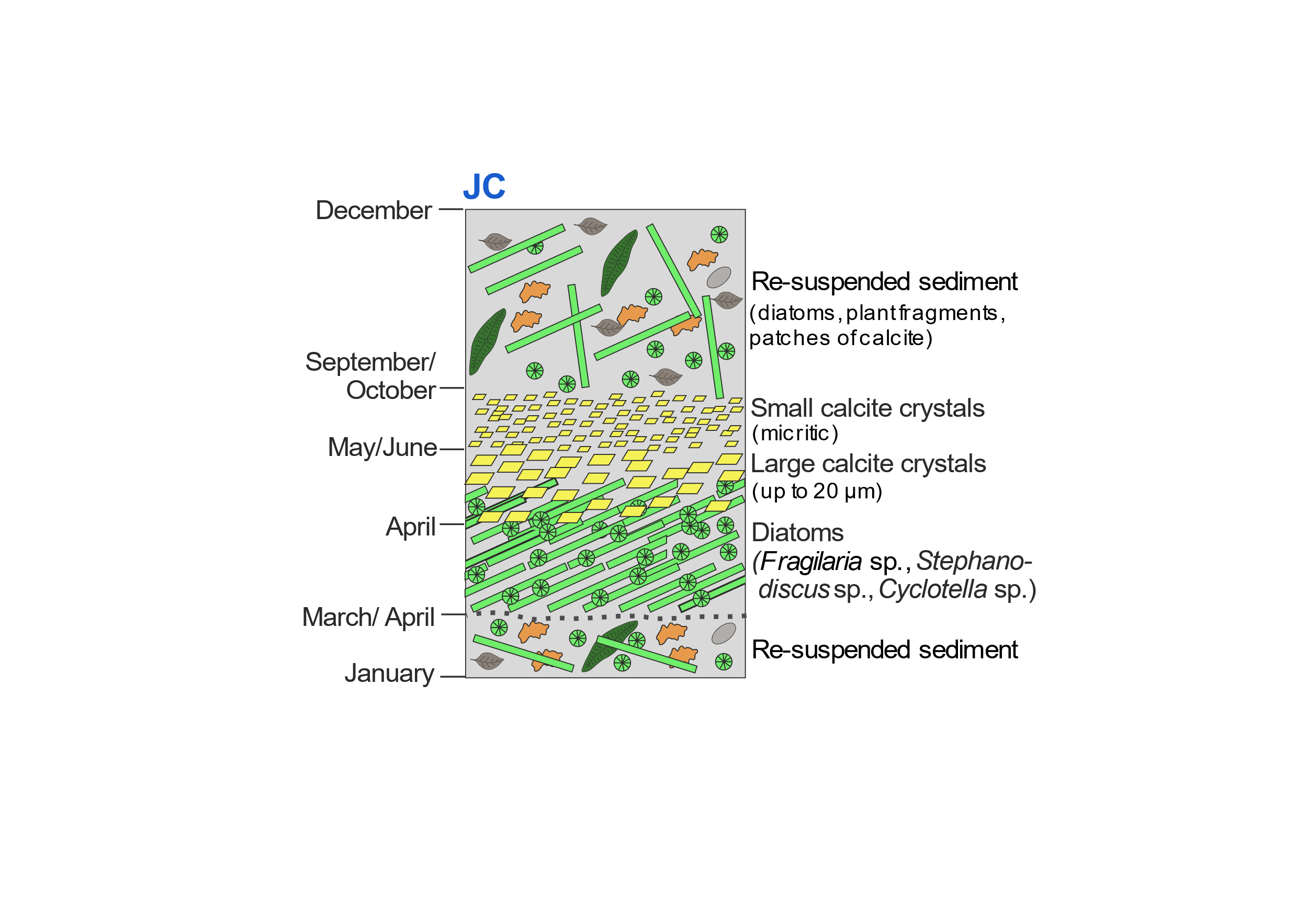


**Fig. S4. Scheme of the seasonal succession of sublayers forming the Lake Czechowskie varves (from Roeser, Dräger ^20^**).


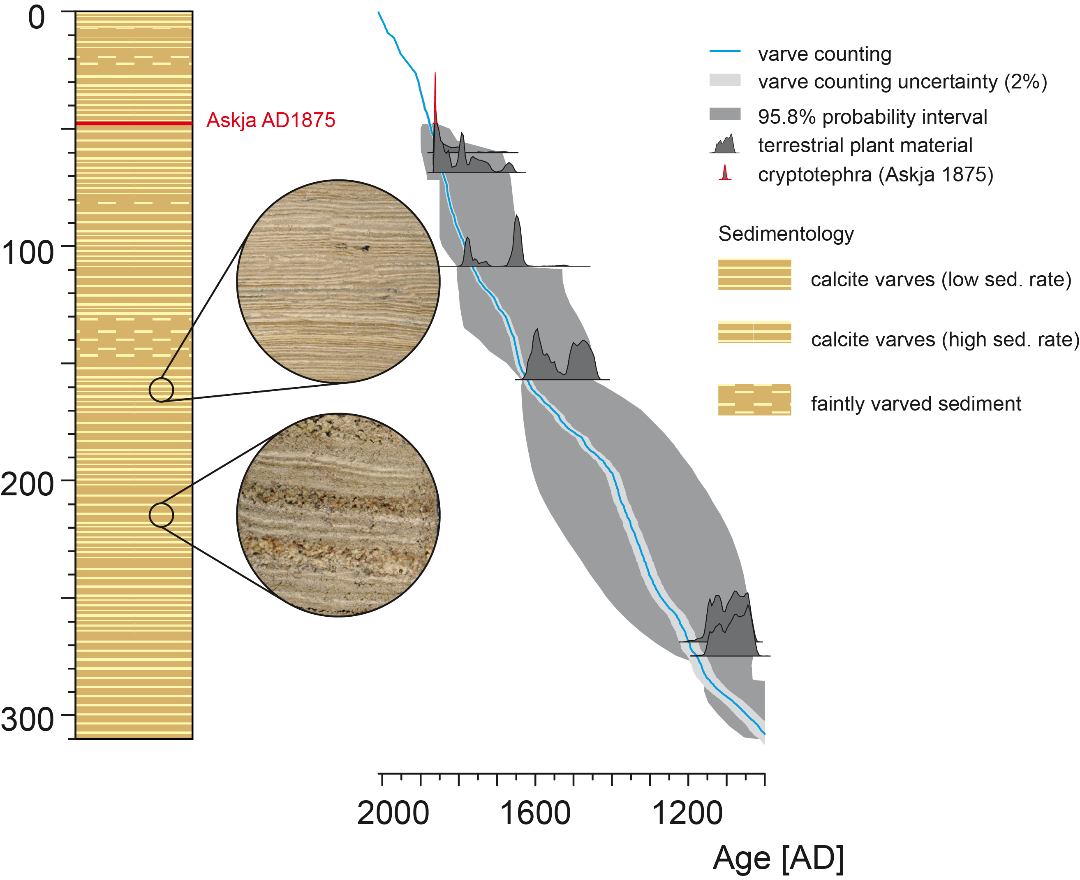


**Fig. S5. Age depth model for the study interval**

**
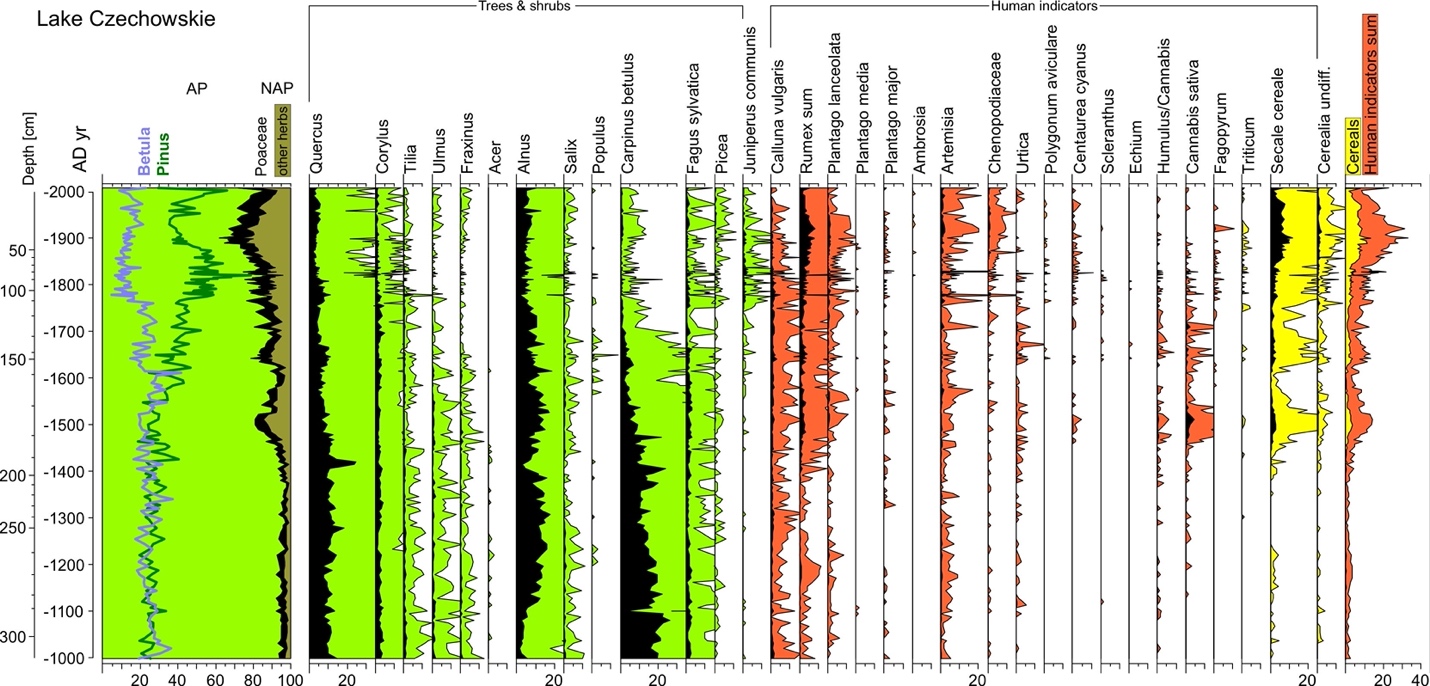
**

**Fig. S6. Selected pollen percentage diagram from the Czechowskie sediment record.**

**Table S1. The amount of the so-called bung tax (Pol. czopowe) paid in 1569 and population in Pomorskie voievodship in 1570**. Data from the tax register summary preserved in: Archiwum Główne Akt Dawnych w Warszawie, Archiwum Skarbu Koronnego I 112. Value ‘–’ means ‘no data’. Population data: Atlas Historyczny Polski. Prusy Królewskie w drugiej połowie XVI wieku, Warszawa: Państwowe Wydawnictwo Naukowe 1961, p.71.

| Tax in 1569 in Polish florins | Settlement name (approx. 1600 AD) | Population in 1570 |
| --- | --- | --- |
| – | Białybór | 654 |
| 634.5 | Chojnice | 1 644 |
| – | Czarne | 720 |
| 26.7 | Człuchów | 510 |
| – | Debrzno | 942 |
| – | Gdańsk | 40 000 |
| 690.4 | Gniew | 966 |
| 92.7 | Kościerzyna | 690 |
| 660.3 | Nowe | 1 094 |
| 408.2 | Puck | 864 |
| 614.8 | Skarszewy | 888 |
| 923 | Starogard | 1 254 |
| 246.8 | Świecie | 1 182 |
| 1 502 | Tczew | 1 290 |
| 599.5 | Tuchola | 1 446 |

**Table S2. A list of villages in the studied area with information about the oldest mention in written sources**. Source: Maksymilian Grzegorz, Osady Pomorza Gdańskiego w latach 1309-1454, Warszawa-Łódź 1990; Maksymilian Grzegorz, Słownik historyczno-geograficzny komturstwa tucholskiego w średniowieczu, Tuchola-Bydgoszcz 2010 (Wydawnictwo Uniwersytetu Kazimierza Wielkiego); Maksymilian Grzegorz, Słownik historyczno-geograficzny komturstwa człuchowskiego w średniowieczu, Bydgoszcz 2016 (Wydawnictwo Uniwersytetu Kazimierza Wielkiego); Karl Kasiske, Das Deutsche Siedelwerk des Mittelalters in Pommerellen, Königsberg  1938 (Kommissionsverlag Gräfe und Unzer).

| Settlement name (HAP database) | First written record mention (year/AD) |
| --- | --- |
| Barchnowy | 1550 |
| Bączek | 1349 |
| Białachowo | 1402 |
| Bietowo | 1402 |
| Bobowo | 1352 |
| Borkowo | 1348 |
| Borzechowo | 1241 |
| Bożepole Szlacheckie | 1360 |
| Bytonia | 1402 |
| Chwarzczenko | 1416 |
| Chwarzno | 1409 |
| Czarnocin | 1378 |
| Czarny | 1395 |
| Czernikowy | 1265 |
| Dąbrowa | 1360 |
| Dolne Maliki | 1319 |
| Garczyn | 1258 |
| Góra | 1402 |
| Górne Maliki | 1319 |
| Grabowo | 1284 |
| Iwiczno | 1438 |
| Jabłowo | 1341 |
| Jaroszewy | 1198 |
| Jezierce | 1350 |
| Kierwałd | 1402 |
| Kleszczewko | 1317 |
| Kobyle | 1258 |
| Kocborowo | 1550 |
| Kokoszki | 1438 |
| Kolińcz | 1323 |
| Koteże | 1401 |
| Kowalikowo | 1371 |
| Koźmin | 1258 |
| Krąg | 1360 |
| Kręski Młyn | 1437 |
| Linowiec | 1335 |
| Lipinki | 1437 |
| Lipy | 1550 |
| Lubichowo | 1352 |
| Łęąg | 1354 |
| Miradowo | 1402 |
| Nowa Wieś | 1437 |
| Osowo | 1402 |
| Osówka | 1437 |
| Owidz | 1340 |
| Pałubin | 1258 |
| Pączewo | 1341 |
| Piesienica | 1402 |
| Pinczyn | 1347 |
| Pogódki | 1258 |
| Radziejewo | 1402 |
| Rokocin | 1349 |
| Rokociński Młyn | 1550 |
| Rombark | 1302 |
| Ropuchy | 1367 |
| Rywałd | 1306 |
| Semlin | 1258 |
| Skórcz | 1274 |
| Smoląg | 1423 |
| Stara Kiszewa | 1269 |
| Stare Polaszki | 1289 |
| Starogard | 1305 |
| Starogard Dwór | 1550 |
| Steklin | 1402 |
| Sucumin | 1402 |
| Sumin | 1284 |
| Wałdówko | 1353 |
| Wda | 1386 |
| Wdecki Młyn | 1550 |
| Wielbrandowo | 1402 |
| Więckowy | 1341 |
| Wolental | 1437 |
| Wolsze | 1328 |
| Wysoka | 1314 |
| Zamek Kiszewski | 1550 |
| Zawada | 1362 |
| Zblewo | 1305 |
| Zelgoszcz | 1352 |
| Zielona Góra | 1373 |
| Złe Mięso | 1550 |
| Żabianki | 1340 |
| Żabno | 1344 |

**Table S3. Number of cultivated fields (in mansi, Pol. łany) at the end of the Middle Ages and in modern times in the studied area**. Value ‘0’ means ‘no data’. All the data comes from the inventories of royal estates in the area surrounding Lake Czechowskie.

| Settlement name (16^th^ century) | Mansi (Middle Ages) | Mansi 1565 AD | Mansi 1624 AD | Mansi 1637 AD | Mansi 1664 AD | Mansi 1765 AD |
| --- | --- | --- | --- | --- | --- | --- |
| Bobowo | 80 | 72 | 76 | 79 | 17 | 79 |
| Borzechowo | 20 | 19 | 20 | 0 | 3 | 3 |
| Bytonia | 40 | 0 | 0 | 40 | 4 | 17,5 |
| Czarny | 60 | 53 | 38 | 0 | 23 | 13,5 |
| Dąbrowa | 60 | 44 | 26 | 60 | 7 | 58 |
| Grabowo | 60 | 54 | 0 | 0 | 21 | 55 |
| Jabłowo | 60 | 64 | 45 | 0 | 14 | 13 |
| Kokoszki | 40 | 60 | 0 | 60 | 0 | 60 |
| Linowiec | 50 | 37 | 3 | 0 | 0 | 0 |
| Lubichowo | 50 | 24 | 23 | 40 | 3 | 11,5 |
| Nowa Wieś | 0 | 20 | 30 | 0 | 10 | 60 |
| Osowo | 30 | 18 | 25 | 0 | 3 | 19 |
| Pączewo | 60 | 40 | 59 | 0 | 27 | 60 |
| Pinczyn | 40 | 27 | 40 | 0 | 10 | 60 |
| Rywałd | 40 | 35 | 0 | 40 | 10 | 40 |
| Semlin | 50 | 23 | 50 | 0 | 0 | 9 |
| Skórcz | 80 | 88 | 0 | 0 | 40 | 88 |
| Wielbrandowo | 50 | 48 | 32 | 0 | 13 | 50 |
| Wolental | 50 | 50 | 47 | 50 | 24 | 50 |
| Wysoka | 60 | 37 | 40 | 60 | 17 | 50 |
| Żabno | 40 | 19 | 3 | 0 | 0 | 0 |
| Zblewo | 50 | 32 | 24 | 50 | 9 | 16 |
| Zelgoszcz | 60 | 48 | 47 | 0 | 22 | 60 |

**Table S4. Radiocarbon dates in study interval (uppermost 300 cm of the Lake Czechowskie sediment record)**

| Lab. Code | Composite depth [cm] | Dated material | AMS 14C age  (yr BP) | Calibrated age  (cal BP, 2 σ error) | Comments | OxCal |
| --- | --- | --- | --- | --- | --- | --- |
| Poz‐38943 | 50.4 | leaf | 165 ± 30 | 143 ± 146 |  | y |
| Poz‐38944 | 60.5 | leaf | 100 ± 30 | 142 ± 127 |  | y |
| Poz‐38942 | 69.1 | leaf | 170 ± 30 | 143 ± 146 |  | y |
| Poz‐52801 | 109 | leaf fragments | 230 ± 30 | 156 ± 158 |  | y |
| Poz‐38939 | 157.5 | leaf | 370 ± 30 | 410 ± 93 |  | y |
| *Poz‐38936* | *256.5* | *leaf* | *990 ± 35* | *880 ± 84* | *omitted* | *n* |
| *Poz‐38931* | *265* | *leaf* | *1065 ± 35* | *991 ± 64* | *omitted* | *n* |
| Poz‐38937 | 269.5 | leaf | 920 ± 30 | 845 ± 77 |  | y |
| Poz‐38938 | 275.5 | Carpinus seed | 930 ± 30 | 854 ± 71 |  | y |

**Table S5. REVEALS parameters: Fall speed of pollen and pollen productivity estimates (PPEs) used for REVEALS application**. PPEs are taken from the PPE.MV2015 data set (PPE for Corylus estimated).

| Taxon | Fall speed | PPE | PPE error |
| --- | --- | --- | --- |
| *Acer* | 0.056 | 1.27 | 0.23 |
| *Alnus* | 0.021 | 16.26 | 1.24 |
| *Betula* | 0.024 | 13.77 | 2.96 |
| *Carpinus* | 0.042 | 3.55 | 0.43 |
| *Corylus* | 0.025 | 10 | 1 |
| *Fagus* | 0.057 | 2.20 | 0.21 |
| *Fraxinus* | 0.022 | 1.07 | 0.31 |
| *Picea* | 0.056 | 0.87 | 0.18 |
| *Pinus* | 0.031 | 4.93 | 0.22 |
| *Quercus* | 0.035 | 9.15 | 1.24 |
| *Tilia* | 0.032 | 2.57 | 2.56 |
| *Ulmus* | 0.032 | 2.60 | 2.55 |
| *Salix* | 0.022 | 1.22 | 0.11 |
| *Artemisia* | 0.025 | 4.56 | 0.51 |
| *Calluna* | 0.038 | 0.82 | 0.02 |
| *Cerealia (excl. Secale)* | 0.060 | 0.15 | 0.02 |
| *Cyperaceae* | 0.035 | 0.87 | 0.06 |
| *Poaceae* | 0.035 | 1 | 0.1 |
| *Plantago lanceolata* | 0.029 | 2.98 | 1.08 |
| *Rumex acetosella* | 0.018 | 3.55 | 0.56 |
| *Secale* | 0.060 | 1.17 | 0.14 |

**References**

1. Słoń M. *Historical atlas of Poland in the 2nd half of the 16th century: voivodeships of Cracow, Sandomierz, Lublin, Sieradz, Łęczyca, Rawa, Płock and Mazovia*. Peter Lang (2014).

2. Chłapowski K, Dygdała J. Prace edytorskie nad lustracjami dóbr królewskich XVI-XVIII w. po półwieczu. *Studia Źródłoznawcze* **43**, 161-171 (2005).

3. Dygdała J. *Lustracja województw Prus Królewskich*. Towarzystwo Naukowe w Toruniu (2003).

4. Biskup M. *Prusy Królewskie w drugiej połowie XVI wieku*. Państwowe Wydawnictwo Naukowe (1961).

5. Mikulski K. *Osadnictwo wiejskie województwa pomorskiego od połowy XVI do końca XVII wieku*. Towarzystwo Naukowe w Toruniu (1994).

6. Siemianowska E, Chudziak W. Problem przyłączenia Pomorza Środkowego do państwa pierwszych Piastów. Głos archeologa. In: *Tradycje i nowoczesność. Początki państwa polskiego na tle środkowoeuropejskim w badaniach interdyscyplinarnych* (eds Kóćka-Krenz H, Matla M, Danielewski M) (2016).

7. Pluskowski A. *The Archaeology of the Prussian Crusade. Holy War and colonisation*. Routledge (2013).

8. Górska-Gołaska K. Margrabska Droga. In: *Słownik historyczno-geograficzny ziem polskich w średniowieczu* (ed Gąsiorowski A) (1993-1999).

9. Szulist W. Ważniejsze szlaki handlowo-komunikacyjne północno-zachodniego Pomorza Gdańskiego w XVI-XVII w. *Zapiski Historyczne* **35**, 105-106 (1970).

10. Wilska M. Roads. In: *Historical atlas of Poland in the 2nd half of the 16th century: voivodeships of Cracow, Sandomierz, Lublin, Sieradz, Łęczyca, Rawa, Płock and Mazovia* (ed Słoń M). Peter Lang (2014).

11. Związek T. Drogi. In: *Wielkopolska w drugiej połowie XVI w.* (eds Chłapowski K, Słoń M). Wydawnictwo Instytutu Historii PAN (2017).

12. Wielopolski A. Polsko-pomorskie spory graniczne w latach 1536-1555. *Przegląd Zachodni* **10**, 85 (1954).

13. Szilágyi M. *On the Road: The History and Archaeology of Medieval Communication Networks in East-Central Europe*. Archaeolingua Alapítvány (2014).

14. Boroda K. *Geografia gospodarcza Królestwa Polskiego w XVI wieku* (2016).

15. Polski AH. Prusy Królewskie w drugiej połowie XVI wieku.). Panstwowe Wydawnictwo Naukowe (1961).

16. Samsonowicz A. Terminologia leśna średniowiecznej Polski. In: *Szkice z dziejów materialnego bytowania społeczeństwa polskiego (Studia i Materiały z Historii Kultury Materialnej)* (ed Dembińska M) (1989).

17. Hoszowski S. *Lustracja województw Prus Królewskich, 1624, z fragmentami lustracji 1615 roku*. Gdańskie Tow. Naukowe (1967).

18. Hoszowski S. Lustracja województwa pomorskiego 1565.). Gdańskie Towarzystwo Naukowe (1961).

19. Brauer A, Casanova J. Chronology and depositional processes of the laminated sediment record from Lac d’Annecy, French Alps*. *Journal of Paleolimnology* **25**, 163-177 (2001).

20. Roeser P*, et al.* Advances in understanding calcite varve formation: new insights from a dual lake monitoring approach in the southern Baltic lowlands. *Boreas*, (2021).

21. Wulf S*, et al.* Holocene tephrostratigraphy of varved sediment records from Lakes Tiefer See (NE Germany) and Czechowskie (N Poland). *Quaternary Science Reviews* **132**, 1-14 (2016).

22. Ott F*, et al.* Site-specific sediment responses to climate change during the last 140 years in three varved lakes in Northern Poland. *The Holocene* **28**, 464-477 (2017).

23. Brauer A*, et al.* The importance of independent chronology in integrating records of past climate change for the 60–8 ka INTIMATE time interval. *Quaternary Science Reviews* **106**, 47-66 (2014).

24. Bronk Ramsey C. Deposition models for chronological records. *Quaternary Science Reviews* **27**, 42-60 (2008).

25. Bronk Ramsey C, Lee S. Recent and Planned Developments of the Program OxCal. *Radiocarbon* **55**, (2013).

26. Tjallingii R, Röhl U, Kölling M, Bickert T. Influence of the water content on X-ray fluorescence core-scanning measurements in soft marine sediments. *Geochemistry, Geophysics, Geosystems* **8**, n/a-n/a (2007).

27. Weltje GJ, Tjallingii R. Calibration of XRF core scanners for quantitative geochemical logging of sediment cores: Theory and application. *Earth and Planetary Science Letters* **274**, 423-438 (2008).

28. Berglund BE, Ralska-Jasiewiczowa M. Pollen analysis. In: *Handbook of Holocene palaeoecology and palaeohydrology* (ed Berglund BE). John Wiley & Sons (1986).

29. Theuerkauf M, Couwenberg J, Kuparinen A, Liebscher V. A matter of dispersal: REVEALSinR introduces state-of-the-art dispersal models to quantitative vegetation reconstruction. *Vegetation History and Archaeobotany*, (2016).

30. Theuerkauf M, Dräger N, Kienel U, Kuparinen A, Brauer A. Effects of changes in land management practices on pollen productivity of open vegetation during the last century derived from varved lake sediments. *The Holocene*, (2015).
